# Knowledge and awareness of medical doctors, medical students and nurses about dentistry in Nigeria

**DOI:** 10.11604/pamj.2016.23.172.7696

**Published:** 2016-04-07

**Authors:** Elijah Olufemi Oyetola, Taiwo Oyewole, Micheal Adedigba, Stephen Tunde Aregbesola, Kehinde Umezudike, Adedotun Adewale

**Affiliations:** 1 Department of Preventive and Community Dentistry, Obafemi Awolowo University Ile-Ife, Nigeria; 2 Department of Oral and Maxillofacial surgery and Oral Pathology, Obafemi Awolowo University, Ile-Ife, Nigeria; 3 Department of Preventive Dentistry, Lagos University Teaching Hospital, Lagos, Nigeria

**Keywords:** Dental awareness, medical students, medical doctors, nurses

## Abstract

**Introduction:**

Various studies have reported poor awareness and knowledge of dentistry in the Nigerian population. There is, however, paucity of information assessing the knowledge and awareness of medical doctors/students and nurses about dentistry. The present study is aimed at determining the knowledge and awareness of medical doctors/students and nurses about dentistry.

**Methods:**

Self-administered questionnaires were randomly distributed among medical doctors/students, and nurses of Obafemi Awolowo Teaching Hospitals’ Complex, Ile-Ife, Nigeria. Information collected using the questionnaire included participants’ biodata, questions evaluating dental awareness, knowledge of systemic and oral health connections as well as referral practices. The data analysis was done with STATA version 11 software.

**Results:**

A total of 300 questionnaires were randomly distributed among doctors/students and nurses, 206 were returned (response rate of 69%). Of the returned questionnaires, 129(63%) were males and 77(37%) were females. There were 42 medical doctors, 49 nurses and 115 medical students. The mean age of the participants was 26.7 years (SD 5.2). Majority (99.5%) was aware of dental profession, but 92% had never referred patients for dental consultation. One third (31%) of medical doctors believed that Ludwig angina was a cardiac disease. A large proportion of the respondents (61%) see no need for routine dental visit while 27% would want to visit the dentist only when they had a dental complaint.

**Conclusion:**

Although a large percentage of the participants claimed to be aware of dentistry, our findings revealed low level of knowledge and attitude to Dentistry. Efforts should be made towards closing this knowledge gap to achieve efficient oral health.

## Introduction

Despite its role in systemic health, oral health care is an aspect that is often neglected [1, 2]. The awareness and knowledge of dentistry is still grossly inadequate among many patients and health care workers [3]. The implication of this is that the vast majority of patients who should benefit from dental services or be referred for their various dental ailments would be unable to assess the services due to the poor awareness and knowledge of dentistry by the attending health care providers [4, 5]. Studies have shown that medical doctors and nurses also have vital roles in oral health care of the population and when awareness is lacking, patients suffer [6, 7]. This becomes necessary due to the well reported relationship between oral health and systemic/general health [8–11]. The need for medical practioners to participate in oral health promotion is highly essential especially in Nigeria where there are limited number of dentist who are unequally distributed [7]. Medical doctors are the primary care givers for the vast majority of health related complaints and they are also expected to play active roles in oral health promotion [12]. As noted earlier, several studies have shown the roles of medical doctors in oral health care delivery. Such roles include the screening for oral diseases, emergency cares, pain management, referral for the alleviation of pain symptoms in general medical practice, pediatrics, and accident and emergency (A and E) department [13]. As such, family physicians and pediatricians have much more important roles to play and when their knowledge of dentistry and oral health care practices are low or poor, inappropriate dental managements and advice or referrals of patients will be the likely outcome. Similar trends were found among medical students in the study by Adeghe et al in which their knowledge of oral health was described as suboptimal as only 2.8% of the 279 medical students had good knowledge of dental specialties [13]. Nurses also play vital roles in health care of patients along with medical doctors [14]. They represent one of the first group of health care providers that give primary care to patients before referral to specialist doctors [15]. Their knowledge of oral health should also be vast so as give appropriate advice when necessary. However, studies have shown that their knowledge about dentistry is quite poor and has been described as been grossly inadequate [15–17]. Some researchers had advocated the need to lay emphasis on dentistry topics during the course of their training [15, 17]. However, Muttineni et al [14] in their study of 147 final year nursing students in India concluded that nursing students had adequate basic knowledge of oral health but were still deficient in correct brushing techniques. Similar findings were also reported by Asif et al [18] amongst nursing students. With the poor awareness of oral health among the medical doctors, nurses and their students in different part of the world, it became necessary to consider this aspect of health care especially in this part of the world where there are limited resources. The aim of the present study was to determine the level of awareness and knowledge of dentistry amongst medical doctors, nurses and medical students in Obafemi Awolowo University Complex Ile Ife Nigeria.

## Methods

### Study design

The study was a descriptive, cross sectional study

### Study location

The study was conducted among the Staff of Obafemi Awolowo University Teaching Hospitals’ Complex (OAUTHC) Ile-Ife. The hospital is located in South Western Nigeria and provides tertiary health care for a teaming population in Osun state as well as the neighboring states of Oyo, Ogun, Ondo, and Kwara. It is a teaching hospital involve in the training of both undergraduate and post graduate medical doctors and dentists. Information was obtained from the participants with self-administered questionnaires. Three hundred questionnaires were distributed and 206 questionnaires were returned correctly filled (response rate of 68.7%), the questionnaires were distributed among Medical doctors, nurses and medical students. The participants were recruited using simple random sampling of the medical doctors, nurses and medical students in the medical and surgical wards.

### Questionnaire design

The questionnaire was designed to have four sections: section A collected information on biodata such as age, sex, marital status and category of the participant such as medical students, nurse or medical doctor; section B contained information on dental awareness and knowledge of dentistry. Some of the questions asked in this section include "Have you ever been to a dentist before?" "What is the specialty of dentistry that treats abnormally arranged teeth? "What are the causes of dental caries?" Section C assessed participants’ knowledge of the relationship between oral health and systemic health. The questions in this aspect assessed participants’ knowledge of Ludwig's angina, periodontal disease and systemic health, nutrition and oral health. Section D evaluated the referral practices of the participants. They were asked when to refer patients with Halitosis, toothache and dental abscess to the dentist.

### Statistical analysis

The information was collected and analyzed using SPSS (Statistical Package for Social Sciences) version 11. Descriptive statistics was used to characterize socio-demographic variables such as age, sex, and marital status. For descriptive continuous variables, the mean, median, minimum value, maximum value and appropriate measures of variability were determined. For descriptive variables that are categorical, simple frequency and percentages was determined.

## Results

### The sociodemographic characteristics of the respondents

A total of 206 subjects participated in the study; 129(63%) males and 77(37%) females. The mean age and standard deviation were 26.7 years and 5.2 respectively with the majority (88.8%) belonging to 21-30 year age group category and 86% had single marital status. More than half (55.8%) of the participants were medical students (Table 1).

**Table 1:** The socio-demographic characteristics of the respondents

	Medical Doctors n = 42 n (%)	Nurses n = 49 n (%)	Medical Student n = 115 n (%)	Total n = 206 n (%)
**Sex**				
Male (%)	33 (78.6)	4 (8.2)	92 (80)	129 (63)
Female (%)	9 (21.4)	45 (91.8)	23 (20)	77 (37)
**Marital Status**				
Married (%)	18 (42.9)	7 (14.3)	3 (2.6)	178 (86)
Single (%)	24 (57.1)	42 (85.7)	112 (97.4)	28 (14)
**Age**(mean = 26.7,SD = 5.2)				
<20 years	0 (0)	0 (0)	1 (0.9)	1 (0.5)
21-30 years	22 (52.4)	47 (96.0)	114 (99.1)	183(88.8)
31-40 years	15 (35.7)	0 (0)	0 (0)	15 (7.3)
41-50 years	5 (11.9)	1 (2.0)	0 (0)	6 (2.9)
51-60 years	0 (0)	1 (2.0)	0 (0)	1 (0.5)

### Dental awareness and attitude of respondents to dentistry

Majority (99.5%) of the respondents was aware of dentistry; the only exception is one medical student who claimed to have never heard about dentistry. Less than half (38%) had been to a dentist for treatment or check up most of whom were medical doctors. Busy work schedules in 91(44%) participants and absence of dental complaints in 97 (47%) participants were some of the reasons why they would not want to seek dental consultation (Table 2).

**Table 2:** Dental awareness and attitude of respondents to dentistry

	Medical Doctors n = 42 (%)	Nurses n = 49	Medical Students n = 115	Total n = 206
**Are you aware of a profession called Dentistry?**				
Yes (%)	42 (100)	49(100)	114 (99.1)	205(99.5)
No (%)	0 (0)	0 (0)	1 (0.9)	1 (0.5)
**Have you ever been to a dentist for treatment or check-up?**				
Yes (%)	29 (69)	12 (24.5)	38 (33)	78 (38)
No (%)	13 (31)	35 (75.5)	77 (67)	127 (62)
**Why would you not want to visit a dentist?**				
Fear of pain (%)	1 (2.4)	3 (6.1)	4 (3.5)	8 (4)
Fear of needle (%)	0 (0)	0 (0)	1 (0.9)	1 (0.5)
High Cost (%)	1 (2.4)	3 (6.1)	3 (2.6)	7 (3.4)
Lack of time (%)	28 (66.7)	12 (24.5)	51 (44.4)	91 (44)
No Dental Complaints (%)	12 (28.6)	31 (63.3)	54 (46.7)	97 (47)
**How often do you brush your teeth?**				
Once Daily	34 (81.0)	31 (63.3)	87 (75.7)	152 (73.5)
Twice Daily	7 (16.8)	18 (36.7)	28 (24.4)	53 (26)
≥Thrice	1 (2.4)	0 (0.0)	0 (0.0)	1 (0.5)
**Appropriate time for routine dental consultations**				
Once every 6 months	7 (6)	0 (0)	7 (17)	14 (7)
Once every year	8 (7)	1 (2)	2 (5)	11 (5)
When there is dental pain/other dental problem	25 (22)	11 (23)	20 (48)	56 (27)
There is no need	75(65)	37 (76)	13 (31)	126 (61)
**Patient with dental abscess:**				
Refer the patient to dental clinic	36 (85.7)	37(75.5)	102 (88.7)	169 (82)
Give antibiotics and analgesics	6 (14.3)	11 (22.5)	12 (10.4)	29 (14)
Ignore/Leave alone	0 (0.0)	1 (2.0)	1 (0.9)	2 (4)
**Patient with toothache:**				
Refer the patient to dental clinic	33 (78.6)	31 (63.3)	96 (83.5)	160 (78)
Give antibiotics and analgesics	9 (21.4)	17 (34.7)	18 (15.6)	44 (21)
Ignore/Leave alone	0 (0.0)	1 (2.0)	1 (0.9)	2 (1)
**Do you think dentists also admit patients?**				
Yes	37 (88.1)	43 (89.6)	85 (73.9)	165 (80)
No	1 (2.4)	3 (6.3)	8 (7.0)	12 (6)
Not Sure	4 (9.5)	3 (6.3)	22 (19.1)	29 (14)

### Respondents’ knowledge about dentistry and dental specialties

Majority (71.4%) of the respondents agreed that Oral and maxillofacial surgery is a dental specialty that treat facial fractures, one medical student representing 0.97% of the respondents’ population attributed management of facial fractures to oral medicine specialty. Less than half of the participants (48.5) were aware that orthodontics treats abnormally arranged teeth. About one-third (32.5%) of the respondents did not know the specialty of dentistry that treats a hole on the tooth and only 14.6% was able to attribute a hole in the tooth to destorative dentistry. Only 23(11%) of the participants associated the management of unusual facial pain to Oral Medicine while the majority of the respondents did not know which of the specialties of dentistry could best manage unusual facial pain (Table 3). Similarly, about two third (61.1%) of the respondents named complex sugar as aetiological factor for dental caries while 9.8% did not know the cause of dental caries.

**Table 3:** Respondents’ knowledge about dentistry and dental specialties

	Medical Doctors n = 42	Nurses n = 49	Medical Students n = 115	Total N = 206
**Specialties of dentistry that treat facial fractures**				
Orthodontics	2 (4.8)	14(28.6)	11 (9.5)	27 (13)
Prosthodontics	0 (0)	4 (8.2)	0 (0)	4 (1.9)
Oral and Maxillofacial Surgery	36 (85.7)	25(51.1)	86 (74.8)	147 (71.4)
Periodontology	2 (4.8)	2 (4.1)	0 (0)	4 (1.9)
Oral Medicine	0 (0)	2 (4.1)	0 (0.0)	2 (0.9)
I don't know	2 (4.8)	2 (4.1)	14 (12.2)	18 (8.7)
Orthodontics & Maxillofacial Surgery	0 (0.0)	0 (0.0)	4 (3.5)	4 (1.9)
Total	42 (100)	49 (100)	115 (100)	206 (100)
**Specialties of dentistry that treat abnormally arranged teeth**				
Orthodontics	26 (61.9)	23(46.9)	51 (44.4)	100 (48.5)
Endodontics	12 (28.6)	13(26.5)	50 (43.5)	75 (36.4)
Maxillofacial Surgery	1 (2.4)	2 (4.1)	4 (3.5)	7 (3.4)
Periodontology	3 (7.1)	10(20.4)	8 (6.7)	21 (10.2)
Oral Medicine	0 (0)	1 (2.0)	1 (0.9)	2 (0.97)
Total	42 (100)	49 (100)	115 (100)	206 (100)
**Specialties of dentistry that treat hole on the tooth**				
Orthodontics	6 (14.3)	5 (10.2)	13 (11.3)	24 (11.7)
Odontodontics	11 (26.2)	14(28.6)	29 (25.2)	54 (26.2)
Maxillofacial Surgery	0 (0)	3 (6.1)	2 (1.8)	5 (2.4)
Periodontologist	3 (7.1)	8 (16.3)	8 (6.7)	19 (9.2)
Oral Medicine	2 (4.8)	3 (6.1)	2 (1.7)	7 (3.4)
Restorative Dentistry	10 (23.8)	8 (16.3)	12 (10.4)	30 (14.6)
I don't know	10 (23.8)	8 (16.3)	49 (42.6)	67 (32.5)
Total	42 (100)	49 (100)	115 (100)	206 (100)
**Specialties of dentistry that treat unusual facial pain**				
Orthodontics	2 (4.8)	4 (8.2)	2 (1.7)	8 (4)
Odontodontics	7 (16.8)	5 (10.2)	10 (8.7)	22 (10)
Maxillofacial Surgery	7 (16.8)	22(44.9)	12 (10.4)	41 (20)
Plastodontics	4 (9.5)	4 (8.2)	12 (10.4)	20 (10)
Oral Medicine	6 (14.3)	5 (10.2)	12 (10.4)	23 (11)
I don't know	16 (38.1)	9 (18.4)	67 (58.3)	92 (45)
Total	42 (100)	49 (100)	115 (100)	206 (100)
**Causes Dental caries**				
Smoking	1 (2.4)	1 (2)	12 (10.4)	14 (6.7)
Complex Sugars	6 (14.3)	13(26.5)	20 (17.4)	39 (18.9)
Bacteria	24 (57.1)	29(59.2)	74 (64.3)	127(61.7)
Tooth picking	10 (23.8)	3 (6.1)	7 (6.1)	20 (9.7)
I don't know	1 (2.4)	3 (6.1)	2 (1.7)	6 (2.9)
Total	42 (100)	49 (100)	115 (100)	206 (100)

### Respondents’ knowledge about systemic and oral health

Only 44(21%) respondents knew Ludwig angina to be a fascial space infection, majority of who were medical doctors. Others either believed it to be a cardiac disease (39.8%) or did not know what it was (35.4%). The Knowledge about periodontal disease exacerbating systemic health; 133(64.5%) of the participant believed periodontitis could exacerbate infective endocarditis. Majority of the respondents believed that oral health could affect the nutrition of patients (Table 4).

**Table 4:** Respondents knowledge about systemic and oral health

	Medical Doctors n = 42	Nurses n = 49	Medical Students n = 115	Total N = 206
**Ludwig's Angina is a:**				
Cardiac Disease	13(31.0)	21 (42.9)	48 (41.7)	82 (39.8)
Renal Disease	0 (0)	1 (2.0)	1 (0.9)	2 (0.97)
Space Infection	21(50.0)	7 (14.3)	16 (13.9)	44 (21.4)
Lung Infection	0 (0)	4 (8.1)	1 (0.9)	5 (2.4)
I don't know	8 (19.1)	16 (32.7)	49 (42.6)	73 (35.4)
**Periodontal disease could exacerbate:**				
Diabetes	0 (0)	2 (4.1)	3 (2.6)	5 (2.4)
Heart Attack	1 (2.4)	0 (0)	1 (0.9)	2 (0.97)
Peptic Ulcer	1 (2.4)	7 (14.3)	0 (0.0)	8 (3.8)
Infective Endocarditis	30(71.4)	30 (61.2)	73 (63.5)	133 (64.5)
I don't know	5 (11.9)	10 (20.4)	35 (30.4)	50 (24.2)
Heart Attack & Infective Endocarditis	0 (0.0)	0 (0.0)	3 (2.6)	3 (1.4)
Diabetes & Infective Endocarditis	5 (11.9)	0 (0.0)	0 (0.0)	5 (2.4)
**A life threatening dental condition:**				
Caries	0 (0.0)	0 (0.0)	1 (0.9)	1 (0.5)
Ludwig's Angina	33(78.6)	24 (49.0)	64 (55.7)	121(58.7)
Periodontal Disease	3 (7.1)	14 (28.6)	6 (5.2)	23 (11.2)
I don't know	5 (11.9)	11 (22.4)	44 (38.3)	60 (29.1)
Ludwig's Angina & Periodontal Disease	1 (2.4)	0 (0.0)	0 (0.0)	1 (0.5)
**Life threatening condition due to untreated dental disease:**				
Brain Tumour	0 (0.0)	1 (2.0)	4 (3.5)	5 (2.4)
Hemangioma	1 (2.4)	8 (16.4)	0 (0.0)	9 (4.4)
Carvenous Thrombosis	34(80.9)	27 (55.1)	57 (49.6)	118 (57.3)
I don't know	7 (16.7)	13 (26.5)	52 (45.2)	72 (35)
**Oral health can affect the nutrition of patients:**				
Yes	42(100.0)	49(100.0)	102(88.7)	193 (93.7)
No	0 (0.0)	0 (0.0)	2 (1.7)	2 (0.97)
Not Sure	0 (0.0)	0 (0.0)	11 (9.6)	11 (5.3)

### Referral practices among respondents

Only about one-third of the repondents strongly agreed that delayed dental treatment could lead to life threatening conditions. A large number (74.4%) also believed that the management of orofacial cancer was best done by oral and maxillofacial Surgeon. Most (92.2%) of the participants had never referred patients for dental consultation. However, majority of them believed that patients with toothache, offensive mouth odor and dental abscess should be referred to dentists for treatment (Table 5).

**Table 5:** Referral practices among respondents

	Medical Doctors	Nurses	Medical Students	Total
**Delayed referal of dental treatments can result in life-threatening condition**				
Strongly agree	15(35.7)	15(30.6)	45 (39.1)	75 (36.4)
Agree	26(61.2)	30(61.2)	57 (49.6)	113 (54.8)
Strongly Disagree	1 (2.4)	2 (4.1)	9 (7.8)	12 (5.8)
Disagree	0 (0)	2 (4.1)	4 (3.5)	6 (2.9)
Total	42 (100)	49 (100)	115 (100)	206 (100)
**Cancers in the orofacial region are best managed by**				
General surgeons	3 (7.1)	10(20.4)	5 (4.4)	18 (8.7)
Maxillofacial surgeons	35(83.3)	34(69.4)	85 (73.9)	154 (74.8)
Plastic surgeons	40(95.2)	1 (2)	4 (3.5)	45 (21.8)
I don't know	0 (0)	0 (0)	16 (13.9)	16 (7.7)
**Have you ever refered any patient to the dentist before**				
Yes	5 (11.9)	1 (2)	11 (9.6)	17 (8.3)
No	37(88.1)	49 (98)	104(90.4)	190 (92.2)
**Patients with toothache**				
Refer to a dentist	33(78.6)	31(62.3)	96 (83.5)	160 (77.7)
Give antibiotic/analgesic	9 (21.4)	17(34.7)	18 (15.6)	44 (21.4)
Ignore	0 (0)	1 (2)	1 (0.9)	2 (0.9)
**Patients with dental abscess**				
Refer to dentist	36(85.7)	37(75.5)	102 (49.5)	175 (84.9)
Give antibiotic/analgesic	9 (21.4)	11(22.4)	12 (10.4)	32 (27.8)
Ignore	0 (0)	1 (2.0)	1 (0.9)	2 (0.9)
**Patients with offensive mouth odour**				
Refer to a dentist	25(59.5)	30(61.2)	99 (86.1)	154 (74.7)
Prescribe mouthwash	10(23.8)	13(26.5)	15 (13.4)	28 (24.3)
Give antibiotic/analgesic	7 (16.7)	6 (12.2)	1 (0.9)	14 (6.7)

## Discussion

Efficient health care delivery is a product of cooperation between every member of the health team [19, 20]. Members of the health team include several groups of health workers, both medical and paramedicals. Smooth interaction is needed between medical and dental team to achieve good health delivery [21]. The present study, designed to determine dental awareness, knowledge and attitude among medical practionners and medical students becomes relevant as findings from this study is expected to throw more light into the importance of maintaning close inter-relationship between medical and dental team. Our study showed that 205 out of 206 participants were aware of dentistry as a profession, representing 95.5% of the study population. This findings is similar to the reports of Chandra et al [22] where 100% of participants of high socio economic status group claimed they are awareness of dental diseases. Similarly, Asif et al [18] also found that 89.9% of their study population knew that there were 32 teeth in the adult cavity. The increased awareness in this study may be due to the fact that the study was done among the educated/professionals. However, results differs in studies done among medical students. Sujatha et al [12] showed that only 25% of the medical students has good oral health awareness. This is not unexpected since the students are in training. Despite the increased oral health campaign in the community at large, it appears that there has not been much of a positive change in attitude and knowledge of people to Dentistry [4, 23]. This was also reflected in our findings that showed only 78(38%) participants had ever visited the dentist for dental checkup/treatment, similar to the report of Bashiru et al [24] who noted that 71.6% of the 360 studied undergraduate students in southern Nigeria never visited a dentist before. However, other studies by Chandral et al [22] reported that 100% of the study population had at least one dental visit in the last one year. Also Doshi et al [25] studied medical students and reported that 79.4% of the participants had visited a dentist for check up at one point or the other in their life. The fear of pain is one of the reasons why people would not want to visit a dentist [2, 22, 26]. However, the present study only reported 4% of participants would not want to visit the dentist for fear of pain. Some of the reasons they gave included busy work schedule (44%) and absence of dental complaints (47%). This is probably because this study was conducted among medical practitioners who are familiar with the hospital environment and probably would have used self medication to control pain [27]. The present study showed that 73.5% of participants brushed their teeth once a day while only 26% of them brushed twice daily. Dhanasekaran et al [28] similarly studied 538 people and noted that only 8.6% of those above the age of 30 years brushed their teeth twice a day while 40% brushed once daily. Some other studies [18, 21, 29] in the scientific literature had shown that most people brush once daily. This may be due to the poor attitude of people to their oral health, busy schedule and poor dental awareness. Unlike Medical/Nursing training in Nigeria where medical/Nursing students had little or no exposure to dental training, training as a dentist involves exposure to basic medical education in the course of their training as Dentists. This must have contributed to why some dentists show basic medical knowledge since medical training was incorporated into their undergraduate curriculum [30]. In the present study we observed gross deficiency in the knowledge of dentistry by medical doctors, nurses and medical students. Only 61.1% of the studied population could associate bacterial to dental caries, 21.4% could attribute Ludwig anginato be a complication of space infection while only 2.4% believed that periodontitis could exacerbate Diabetes mellitus. We also noted that medical doctors had a better knowledge compared to others. However, the knowledge is still unacceptable and this showed a gross lack of basic dental knowledge among medical health workers as earlier reported by many researchers [13, 31–33]. This is largely due to the little or no exposure to dental education during the course of their training. Therefore, inclusion of dental education into the medical curriculum will go a long way in improving dental knowledge and skills among medical practitioners as suggested by many studies [18, 21, 31, 33]. Oral health is an integral part of systemic health. Adequate level of understanding and cooperation is therefore needed between medical and dental health teams to ensure qualitative health care delivery. In addition, acquisition of basic knowledge of dentistry by medical health workers will go a long way in improving health care especially in the developing world with limited human resources.

## Conclusion

This study has shown that majority of health workers within the hospital are aware of the dental profession but their knowledge and attitude to dentistry is still poor. Their oral hygiene practices in which majority brushed one daily and large number of them never visiting the dental clinic for check up and never refer patients for dental consultation are points to elaborate the need for understanding between medical and dental profession. Our study also demonstrated that medical doctors were better informed about Dentistry when compared to Nurses and Medical students. Inclusion of dental education at both undergraduate and postgraduate medical curriculum appear to be a promising solution to this unnecessary knowledge gap between the medical and dental profession.

### What is known about this topic

- Generally there is poor awareness and knowledge of dentistry among the populace especially in the developing part of the world;
- Health workers are also included but little is known about the actual level of awareness of the various categories of health workers such as medical doctors/students and nurses.

### What this study adds

- The present study determined the level of awareness and knowledge of dentistry amongst medical doctors, nurses and medical students in Obafemi Awolowo University Complex Ile Ife Nigeria.
